# Human Papillomavirus Infection Across the Immunological Spectrum: Clinical Expression, Colposcopic Challenges, and Therapeutic Implications

**DOI:** 10.3390/diagnostics16121932

**Published:** 2026-06-22

**Authors:** Antonio Braga, Gustavo Ribeiro Lima, Karine Mello Duvivier, Edward Araujo Júnior, Caroline Alves de Oliveira Martins, Isabel Cristina Chulvis do Val Guimarães, Susana Cristina Aidé Viviani Fialho

**Affiliations:** 1 Department of Gynecology and Obstetrics, School of Medicine, Federal University of Rio de Janeiro (UFRJ), Rio de Janeiro 22240-003, RJ, Brazil; 2 Department of General and Specialized Surgery, School of Medicine and Surgery, Federal University of the State of Rio de Janeiro (UNIRIO), Rio de Janeiro 22290-240, RJ, Brazil; 3 Postgraduate Program in Applied Health Sciences, University of Vassouras (Univassouras), Vassouras 27700-000, RJ, Brazil; 4 Service of Gynecology and Obstetrics, Antônio Pedro University Hospital, Fluminense Federal University (UFF), Niterói 24070-090, RJ, Brazil; gustavoribeirolima@hotmail.com (G.R.L.); karineduvivier@id.uff.br (K.M.D.); carolineaom@id.uff.br (C.A.d.O.M.); isabelval@id.uff.br (I.C.C.d.V.G.); susanaaide@id.uff.br (S.C.A.V.F.); 5 Discipline of Woman Health, Municipal University of São Caetano do Sul (USCS), São Caetano do Sul 09521-160, SP, Brazil; araujojred@terra.com.br; 6 Department of Obstetrics, Paulista School of Medicine, Federal University of São Paulo (EPM-UNIFESP), São Paulo 04023-062, SP, Brazil; 7 Department of Maternal and Child Health, School of Medicine, Fluminense Federal University (UFF), Niterói 24070-090, RJ, Brazil

**Keywords:** human papillomavirus, immunosuppression, cervical intraepithelial neoplasia, colposcopy, vaccination

## Abstract

Human papillomavirus (HPV) infection is a major driver of anogenital disease and virus-related carcinogenesis. Although most infections resolve spontaneously, persistent infection with high-risk genotypes may progress to high-grade squamous intraepithelial lesions (HSILs) and cancer, particularly in the setting of impaired immune surveillance. Unlike previous HPV-related reviews focused primarily on cervical disease, vaccination, or isolated immunosuppressed populations, this narrative review comparatively examines the clinical expression, colposcopic findings, screening strategies, and therapeutic implications of HPV-related disease across the immunological spectrum. This narrative review provides an integrative synthesis of HPV-related disease in the female lower genital tract across the immunological spectrum. A structured, non-systematic search of PubMed/MEDLINE, Scopus, and Web of Science was conducted using terms related to “human papillomavirus”, “HPV”, “cervical intraepithelial neoplasia”, “colposcopy”, “immunosuppression”, “HIV”, and “vaccination”. Immunosuppressed populations, including individuals living with HIV, transplant recipients, and patients receiving immunosuppressive therapy, exhibit higher rates of persistent infection, multifocal disease, recurrence, and progression to HSIL and invasive malignancy. These patients also present greater diagnostic complexity, broader anatomical involvement, and reduced response to conventional treatment. Rather than representing a uniform condition, HPV-related disease reflects a biologically dynamic spectrum shaped by host immune competence. This review highlights the distinct clinical, colposcopic, and therapeutic challenges observed in immunosuppressed populations and reinforces the need for individualized, risk-adapted strategies integrating contemporary advances in screening, vaccination, and HPV-related disease management.

## 1. Introduction

Human papillomavirus (HPV) infection represents one of the most prevalent sexually transmitted infections worldwide and a central model of virus-driven carcinogenesis. It is associated with a broad clinical spectrum ranging from transient, self-limited lesions to invasive malignancies affecting multiple anogenital sites [1,2,3]. Globally, HPV was responsible for approximately 831,000 new cancer cases and 423,000 deaths in 2022, accounting for about 4.5–5% of all cancers worldwide, with a disproportionate impact on women [4,5,6,7,8]. This burden reflects not only the biological relevance of HPV but also persistent inequities in access to vaccination, screening, and timely treatment.

Although most HPV infections are transient and spontaneously cleared, persistent infection with high-risk genotypes is the key event associated with progression to high-grade intraepithelial lesions and invasive malignancies [9,10,11,12,13,14,15]. Cervical cancer remains the most prominent HPV-related malignancy, particularly in low- and middle-income countries, despite advances in vaccination and screening programs [4,5,6,7,8].

Host immune competence plays a central role in shaping the natural history of HPV infection. In immunosuppressed populations, including individuals living with as human immunodeficiency virus (HIV), transplant recipients, and patients receiving immunosuppressive therapy, HPV-related disease is characterized by higher rates of viral persistence, multifocal lower genital tract involvement, recurrence, and progression to malignancy [16,17,18,19,20,21]. In these settings, diagnostic evaluation and therapeutic management are frequently more complex, often requiring broader anatomical assessment and prolonged surveillance [21,22,23,24].

Despite growing recognition of these differences, important gaps remain regarding optimal screening, diagnostic, and therapeutic strategies across distinct immunological contexts. Beyond its well-established role in the development of anogenital neoplasia and cancer, HPV infection may also exert a substantial psychosocial burden on affected individuals. Similar to other sexually transmitted infections, HPV-related disease has been associated with increased levels of anxiety, distress, stigma, and concerns regarding transmissibility and future health, as well as negative effects on body image, sexual functioning, and overall quality of life. These consequences may be particularly pronounced in women requiring repeated surveillance, colposcopic evaluations, or treatment for persistent and recurrent disease. Therefore, the impact of HPV extends beyond its biological and oncogenic effects, encompassing important psychological, relational, and sexual health dimensions that should be considered in comprehensive patient care.

In this context, this narrative review differs from previous HPV-related review articles by providing an integrated and comparative analysis of HPV infection across the entire immunological spectrum, rather than focusing exclusively on cervical disease, vaccination, or isolated immunosuppressed populations. This narrative review aims to comparatively examine the clinical, colposcopic, and therapeutic aspects of HPV infection in the female genital tract among immunocompetent and immunosuppressed women.

By framing HPV-related disease along a continuum defined by immune competence, this review seeks to support a more individualized and biologically informed approach to care. In doing so, it aims to contribute to improved clinical decision-making, inform future guideline development, and align with global efforts to reduce the burden of HPV-associated neoplasia, particularly among populations at highest risk.

## 2. Methods

This narrative review was conducted to provide a clinically oriented and integrative synthesis of current evidence regarding HPV infection in the female genital tract, with emphasis on differences between immunocompetent and immunosuppressed populations. The manuscript was developed through the qualitative integration of previously published studies, contemporary evidence, and international guidelines.

A structured, yet non-systematic, literature search was conducted across major biomedical databases, including PubMed/MEDLINE, Scopus, and Web of Science, to identify relevant publications. The search strategy combined controlled vocabulary and free-text terms related to “human papillomavirus”, “HPV”, “cervical intraepithelial neoplasia”, “colposcopy”, “immunosuppression”, “HIV”, and “vaccination”. Additional references were identified through manual review of selected articles and key review papers.

The selection of studies prioritized recent evidence, high-quality observational studies, clinical trials, systematic reviews, and meta-analyses, as well as international guidelines and consensus statements, including those from organizations such as the World Health Organization (WHO), American Society for Colposcopy and Cervical Pathology (ASCCP), American College of Obstetricians and Gynecologists (ACOG), National Comprehensive Cancer Network (NCCN), International Federation for Cervical Pathology and Colposcopy (IFCPC), and HIV-specific guideline panels. Classic and landmark studies were also included when essential to contextualize the evolution of concepts related to HPV natural history and immunology.

Given the narrative nature of this review, no formal inclusion or exclusion criteria, quality scoring system, or meta-analytic statistical methods were applied. Instead, articles were selected based on their relevance, methodological robustness, and contribution to the conceptual understanding of the topic. Particular emphasis was placed on studies addressing clinical manifestations, colposcopic findings, screening strategies, diagnostic tools, therapeutic approaches, and outcomes in both immunocompetent and immunocompromised populations.

The synthesis of evidence was performed qualitatively, with integration of findings across different domains, including virology, immunology, epidemiology, and clinical practice. When appropriate, differences and similarities between populations were highlighted to support a comparative framework throughout the manuscript. The structure of the review was predefined to follow key thematic domains: definitions and population scope, natural history and immunology, clinical manifestations by anatomical site, colposcopic assessment, screening and diagnostic strategies, vaccination, and treatment, ensuring internal coherence and alignment with the proposed objectives.

Although this review does not adhere to a systematic review protocol, efforts were made to enhance transparency and reproducibility by clearly describing the search approach, sources of evidence, and rationale for study selection. This methodological choice reflects the intent to provide a broad, integrative, and clinically meaningful overview, rather than a quantitative synthesis, thereby supporting informed decision-making in complex and heterogeneous clinical scenarios.

## 3. Conceptual Framework and Population Stratification

HPV is a double-stranded DNA virus belonging to the *Papillomaviridae* family, characterized by remarkable genotypic diversity. More than 450 genotypes have been identified, of which approximately 40 exhibit tropism for the anogenital mucosa [25]. These genotypes are classified according to their oncogenic potential into high-risk and low-risk categories. High-risk types, including HPV16, HPV18, HPV31, HPV33, HPV45, HPV52, and HPV58, are responsible for the vast majority of HPV-related malignancies, whereas low-risk types such as HPV6 and HPV11 are primarily associated with benign lesions, particularly anogenital warts [3,26].

The biological distinction between these groups is grounded in their molecular behavior, reflecting fundamental differences in viral biology, particularly in their ability to integrate into the host genome and to induce genetic instability—key mechanisms directly associated with viral persistence and progression to high-grade intraepithelial lesions and invasive carcinoma [26]. High-risk genotypes express the viral oncoproteins E6 and E7, which promote degradation of tumor suppressor proteins p53 and pRb, leading to cell cycle dysregulation, genomic instability, and malignant transformation [27]. In contrast, low-risk types typically maintain their genome in episomal form and have limited capacity to interfere with host cell regulatory pathways, explaining their predominantly benign clinical course [28,29].

From a clinical perspective, the outcome of HPV infection is largely determined by host immune competence. In immunocompetent individuals, effective cell-mediated immunity leads to viral clearance in most cases, usually within 2 years [30,31]. This response is primarily driven by CD4+ T helper cells and CD8+ cytotoxic T lymphocytes, which coordinate the elimination of infected epithelial cells. As a result, most infections remain transient and clinically silent.

In contrast, immunosuppressed individuals represent a heterogeneous group in whom this balance is disrupted [12]. This population includes individuals living with HIV, recipients of solid organ or hematopoietic stem cell transplantation, patients undergoing long-term immunosuppressive or biological therapies, and those with primary immunodeficiencies. In these settings, impaired cellular immunity results in reduced viral clearance, increased susceptibility to persistent infection, and a higher likelihood of infection with multiple HPV genotypes simultaneously [20].

Importantly, immunosuppression should not be viewed as a binary condition but rather as a spectrum, in which the degree and duration of immune dysfunction directly influence the natural history of HPV infection. More profound immunosuppression, particularly when associated with significant CD4+ T-cell depletion, is consistently associated with higher rates of persistence, progression to HSIL, and development of invasive malignancies.

This risk gradient is particularly evident in people living with HIV, in whom HPV-related disease remains elevated despite antiretroviral therapy [32]. The association is strongest at CD4 counts < 200–350 cells/µL, with significantly higher rates of high-grade intraepithelial lesions and invasive cervical cancer [33]. Although ART improves survival, its impact on HPV natural history is limited and does not fully restore immune control. Notably, HPV16 shows relatively stable prevalence regardless of immunosuppression, unlike other genotypes that increase as CD4 declines, suggesting greater immune evasion and explaining its high oncogenicity [32,34]. In vaccinated populations, high-grade cervical lesions decline, accompanied by reduced cytology positive predictive value, requiring adaptation of screening programs [35].

In addition, women living with HIV exhibit a high prevalence of multiple HPV infections, often involving several high-risk genotypes simultaneously, which is associated with an increased risk of precursor lesions and neoplastic. This viral multiplicity reflects both increased exposure and an impaired capacity of the immune system to effectively clear distinct viral types [32,36].

Non-HIV-related immunosuppression encompasses a heterogeneous group of clinical conditions, including solid organ transplant recipients, hematopoietic stem cell transplant recipients, and patients receiving long-term immunosuppressive therapy for autoimmune diseases. In these settings, the risk of persistent HPV infection and the development of HPV-associated neoplasms is significantly increased, varying according to the intensity and duration of immunosuppression. In solid organ transplant recipients, there is a marked increase in HPV-related cancers, including cervical, anal, vulvar, vaginal, penile, and oropharyngeal, due to chronic iatrogenic immunosuppression. This impairs immune control of HPV, leading to higher incidence and faster progression. Cancer risk is dynamic, influenced by the intensity and duration of immunosuppression and the transplanted organ type [37].

Patients with autoimmune diseases undergoing immunosuppressive therapy also demonstrate an increased risk of cervical dysplasia and cancer, although the magnitude of this risk varies depending on the underlying condition and the specific therapeutic regimen [38]. In addition, individuals with primary immunodeficiencies show heightened susceptibility to infection with multiple HPV genotypes, as well as the development of extensive and recurrent lesions [39].

Despite differences in etiology, immunosuppressed populations share a set of defining clinical features: increased prevalence of high-risk genotypes, greater frequency of multiple concurrent infections, higher rates of multifocal disease involving the cervix, vagina, vulva, and anal canal, and a markedly elevated risk of recurrence following treatment [12,20,36,39,40]. These characteristics reflect the inability of the host immune system to control viral replication and limit epithelial spread effectively [29].

The recognition of these patterns is fundamental for clinical practice. It supports the need for a risk-adapted approach to screening, diagnosis, and treatment and provides the conceptual basis for comparing HPV-related disease across different immunological contexts [24]. In this review, populations are therefore stratified according to immunological status, allowing a structured analysis of how host immunity shapes the clinical expression, diagnostic complexity, and therapeutic outcomes of HPV infection. Rather than representing distinct categories, immunocompetent and immunosuppressed states define a continuum in which the degree of immune dysfunction directly shapes the clinical expression, persistence, and oncogenic potential of HPV infection.

**Figure 1:**
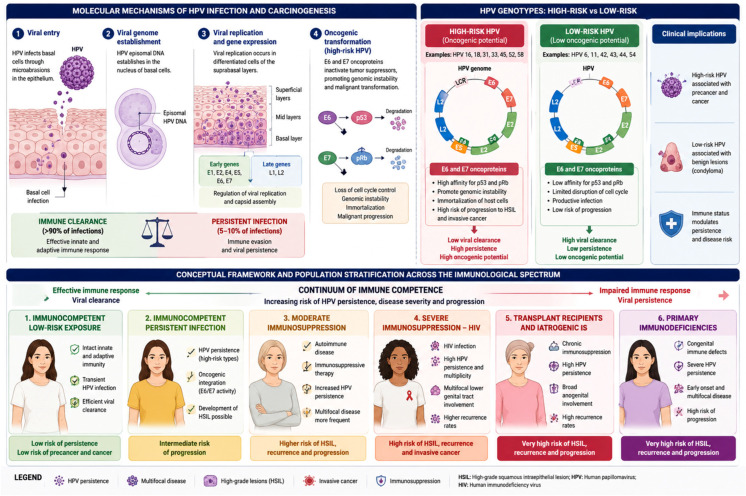
Molecular mechanisms of HPV infection and carcinogenesis across the immunological spectrum, highlighting the differences between high-risk and low-risk HPV genotypes and the impact of host immune competence on viral persistence, multifocal disease, and progression to HSIL and invasive malignancy.

To facilitate understanding of the biological basis of HPV-related disease across different immunological contexts, Figure 1 summarizes the molecular mechanisms involved in HPV infection, persistence, and carcinogenesis. The figure also illustrates the distinct biological behavior of high-risk and low-risk HPV genotypes, highlighting differences in viral oncogenic potential, host immune interaction, persistence, and progression to intraepithelial lesions and malignancy. In addition, the schematic integrates the concept of the immunological spectrum, demonstrating how varying degrees of immune competence influence disease expression, multifocality, and clinical outcomes.

## 4. Natural History and Immunology

HPV infection follows a dynamic and multi-step course characterized by viral acquisition, replication within the basal epithelial layer, potential persistence, and, in a minority of cases, progression to precancerous lesions and invasive malignancy. Acquisition typically occurs shortly after the onset of sexual activity, reflecting the high transmissibility of the virus, with approximately 38.9% of women acquiring HPV infection within 24 months of their first sexual intercourse [30,41]. Despite this, most infections remain asymptomatic and transient, reflecting effective immune control, with spontaneous viral clearance occurring in the majority of individuals within 12 to 24 months, particularly in immunocompetent women.

The temporal dynamics of HPV infection are influenced by both viral and host factors. After initial infection, viral replication reaches a plateau and may persist at low levels before declining as a result of host immune responses [42]. While the median duration of infection is relatively short, a subset of infections, particularly those caused by high-risk genotypes such as HPV16, demonstrates a greater propensity for persistence [43]. This persistence represents the central event in HPV-related carcinogenesis, as it allows sustained expression of viral oncoproteins and progressive accumulation of genetic and epigenetic alterations in the host epithelium [41].

Persistent infection with the same genotype is the strongest predictor of progression to high-grade squamous intraepithelial lesions (HSILs) and invasive cancer [31]. This process is typically slow and may take years or even decades, involving a transition from productive infection to transforming infection [43,44]. During this phase, viral deoxyribonucleic acid (DNA) may integrate into the host genome, leading to deregulated expression of E6 and E7, disruption of cell cycle control, and genomic instability. Importantly, not all persistent infections progress, and spontaneous regression remains possible, reflecting the ongoing interplay between viral activity and host immune surveillance [45].

Cell-mediated immunity plays a central role in determining the outcome of HPV infection. Effective immune responses are largely driven by CD4+ T helper cells, particularly Th1-type responses, which orchestrate the activation of cytotoxic CD8+ T lymphocytes [12]. These effector cells recognize and eliminate infected keratinocytes, contributing to viral clearance and lesion regression [46]. Histopathological studies consistently demonstrate that regressing lesions are characterized by dense lymphocytic infiltrates, whereas persistent or progressive lesions exhibit a more permissive immune microenvironment, often enriched with regulatory T-cells and reduced cytotoxic activity [45].

HPV has evolved multiple mechanisms to evade immune detection and clearance. The infection is non-lytic and confined to the epithelium, minimizing the release of inflammatory signals that would otherwise activate innate immunity [12]. In addition, the virus limits antigen presentation in basal epithelial cells and interferes with interferon signaling pathways, thereby impairing both innate and adaptive immune responses [29]. This immune evasion strategy enables the virus to establish chronic infection, particularly in hosts with compromised immune function.

The balance between viral clearance and persistence is modulated by several host-related factors, including age, hormonal milieu, smoking, and co-infections [41,43,47]. However, immunological status remains the most critical determinant. In immunocompetent individuals, the immune system is generally capable of controlling viral replication and preventing long-term persistence. In contrast, immunosuppression profoundly alters this equilibrium, shifting natural history toward chronic infection and increased oncogenic risk.

In individuals living with HIV, reduced CD4+ T-cell counts are strongly associated with higher rates of HPV acquisition, persistence, and progression [33,34]. Even in the context of effective antiretroviral therapy, immune reconstitution is often incomplete, and the risk of HPV-related disease remains elevated [32,36]. Similarly, in transplant recipients and patients receiving long-term immunosuppressive therapy, impaired immune surveillance facilitates persistent infection, increased viral load, and the development of multifocal lesions across the anogenital tract [37].

A hallmark of HPV infection in immunosuppressed populations is the increased frequency of infection with multiple genotypes simultaneously, including high-risk types. This multiplicity reflects both increased exposure and diminished immune control, and it is associated with a higher likelihood of progression to HSIL and invasive disease. In addition, lesions in these individuals tend to be more extensive, more rapidly progressive, and more likely to recur after treatment, underscoring the clinical relevance of immune status in shaping disease behavior.

The integration of immunological and virological processes provides a comprehensive framework for understanding HPV-related carcinogenesis. Persistent viral infection, coupled with ineffective immune surveillance and active immune evasion, creates a permissive environment for malignant transformation. This process is markedly accelerated in the setting of immunosuppression, where the loss of immune control allows sustained viral activity and progressive epithelial damage [27,37,44].

Understanding these processes is essential for developing effective prevention, screening, and treatment strategies. It further supports tailored clinical approaches based on immunological status, as HPV infection follows a continuum shaped by host–virus interactions. This framework explains the distinct clinical patterns observed across the immunological spectrum, linking viral persistence, immune evasion, and disease progression.

## 5. Clinical Manifestations by Anatomical Site

The clinical expression of HPV infection in the female lower genital tract encompasses a wide spectrum of lesions, ranging from benign proliferative conditions to high-grade precancerous disease. These manifestations are strongly influenced by viral genotype and host immune status and exhibit distinct patterns according to the anatomical site involved. Importantly, HPV-related disease frequently demonstrates multifocal distribution, requiring systematic evaluation of the entire anogenital tract.

### 5.1. Cervix

The cervix represents the most extensively studied site of HPV-related disease and remains the primary target of screening strategies. Cervical manifestations encompass a broad spectrum, ranging from benign lesions to precursor changes in malignancy, reflecting the interaction between viral genotype and host immune response. These manifestations include subclinical infection, cervical condylomas, low-grade squamous intraepithelial lesions (LSILs), and HSILs, which correspond to increasing degrees of epithelial dysplasia and risk of progression to invasive carcinoma.

Benign manifestations, such as cervical condylomas, although less common than in other anatomical sites, may occur and are typically associated with low-risk HPV genotypes, particularly HPV6 and HPV11, which are responsible for the majority of anogenital warts [9]. These lesions may present as exophytic or flat growths and are often asymptomatic, being identified incidentally during colposcopic examination. Colposcopically, they appear as subtle acetowhite areas with minimal vascular changes, which may hinder recognition, especially when coexisting with other epithelial abnormalities [48]. Despite their benign nature, recurrence is common, particularly in the context of coinfection or immunosuppression, and these lesions may coexist with intraepithelial lesions of higher grade [9].

LSIL typically reflects productive viral infection and is associated with high rates of spontaneous regression, particularly in younger and immunocompetent individuals. In contrast, HSIL represents transforming infection driven by persistent high-risk HPV and constitutes the immediate precursor to cervical cancer [24,44]. Clinically, these lesions are predominantly asymptomatic and are most often detected through screening with cytology or HPV testing, followed by colposcopic evaluation and histopathological confirmation, reinforcing the importance of organized prevention programs.

**Figure 2:**
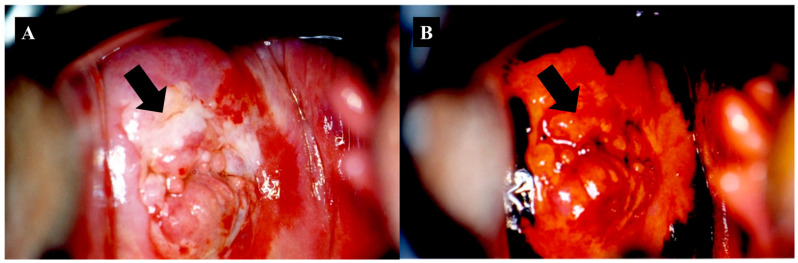
Colposcopic findings in a 42-year-old woman living with HIV. (**A**) Colposcopy after application of acetic acid demonstrating dense acetowhite epithelium (black arrow), consistent with a major colposcopic abnormality suggestive of HSIL. (**B**) Colposcopy following the Schiller test showing iodine-negative epithelium (black arrow; Schiller-positive), reinforcing the suspicion of high-grade disease.

In addition to the classic spectrum described above, colposcopic evaluation remains essential for identifying high-grade lesions, particularly in patients with altered immune status, in whom disease may present with more extensive and atypical features. Figure 2 illustrates representative colposcopic features of a high-grade lesion in a patient living with HIV. Dense acetowhite epithelium following acetic acid application, together with iodine negativity on the Schiller test, supports the identification of a major abnormality consistent with HSIL and reinforces the need for histopathological confirmation.

### 5.2. Vagina

Vaginal involvement, typically referred to as vaginal intraepithelial neoplasia, represents a less frequent but clinically relevant manifestation of HPV infection. Vaginal lesions are often identified in patients with prior or concurrent cervical disease, reflecting persistent viral infection within the lower genital tract [49].

**Figure 3:**
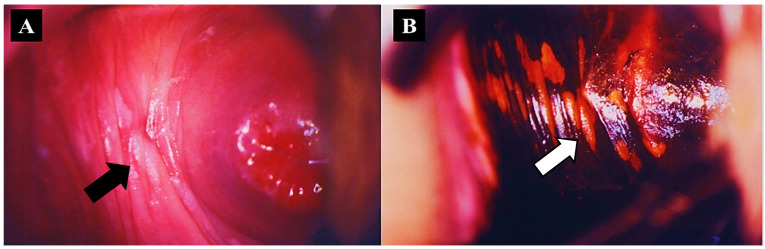
(**A**) Vagina after acetic acid application showing dense acetowhite epithelium with multifocality (black arrow). (**B**) Vagina following the Schiller test demonstrating iodine-negative epithelium with multifocality (white arrow).

Clinically, these lesions are usually asymptomatic and detected during colposcopic examination [50]. They often present as acetowhite areas located in the upper vagina, particularly at the vaginal cuff in women who have undergone hysterectomy [49]. A key feature of vaginal disease is its strong association with multifocality, frequently coexisting with cervical or vulvar lesions, consistent with the concept of a field effect in HPV infection, as shown in Figure 3 [50].

### 5.3. Vulva

The vulva is a common site of both benign and premalignant HPV-related lesions. Anogenital warts (condyloma acuminata) are the most frequent manifestation and are primarily associated with low-risk HPV types [9]. These lesions present as papular, flat, or exophytic growths, often involving the labia, perineum, and perianal region. Although benign, they may cause discomfort, pruritus, and significant psychosocial distress [48].

In contrast, vulvar HSIL represents the premalignant form associated with high-risk HPV infection [51,52]. These lesions typically occur in younger women and may present with variable morphology, including plaques, papules, or areas of discoloration. Clinical recognition may be challenging due to heterogeneity in appearance, requiring a high index of suspicion and histopathological confirmation.

**Figure 4:**
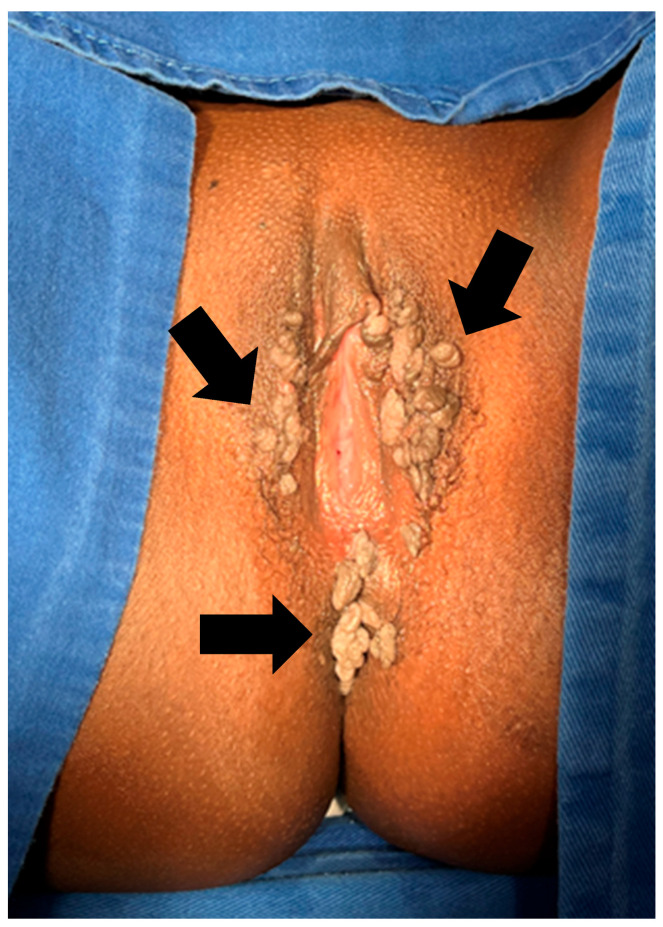
Extensive anogenital warts in an immunosuppressed patient. Vulvar and perianal condyloma acuminata (black arrows) in a 37-year-old woman with systemic lupus erythematosus, illustrating the multifocal and extensive pattern of HPV-related disease in the setting of immune dysfunction.

In the context of impaired immune competence, vulvar HPV-related disease often presents with greater lesion burden, multifocal distribution, and increased recurrence rates, reflecting reduced viral clearance [53]. This pattern is not limited to HIV infection and may also be observed in other forms of immunosuppression. As illustrated in Figure 4, extensive anogenital warts involving the vulvar and perianal regions exemplify the clinical expression of HPV infection in this immunologically altered setting, reinforcing the need for individualized management and closer surveillance.

**Figure 5:**
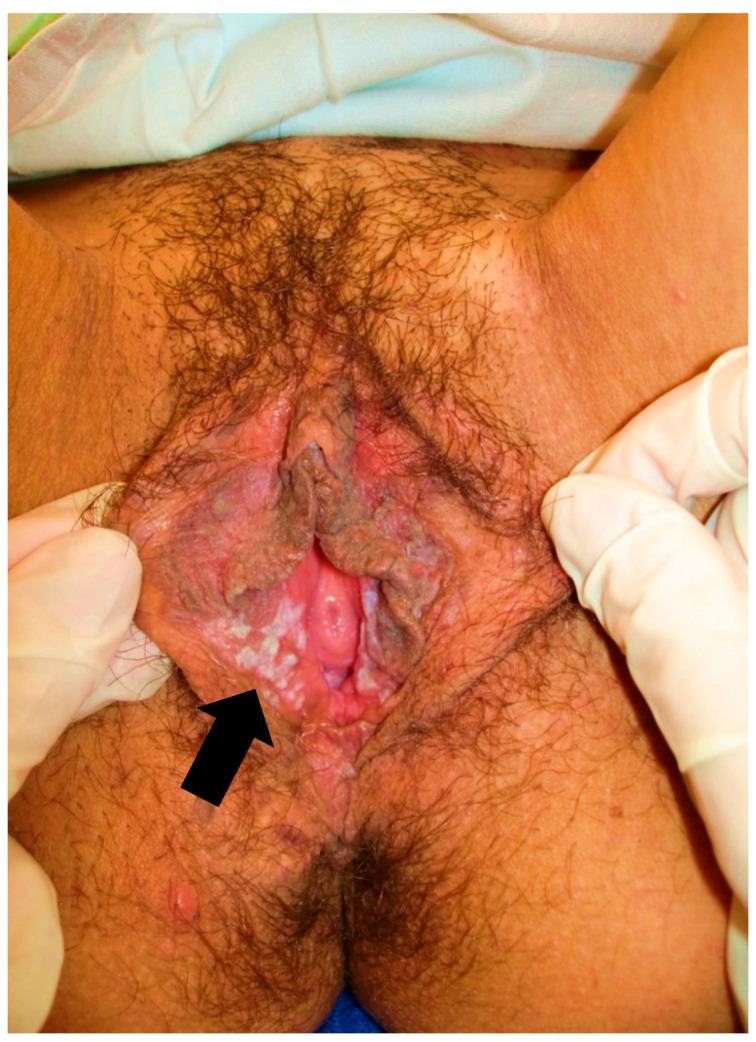
Complex vulvar HPV-related lesions in an immunosuppressed patient. Extensive, confluent vulvar lesions with irregular surface, hyperkeratosis, and areas of pigmentation (black arrow), illustrating the heterogeneous morphology of HPV-related disease in the setting of immune dysfunction and the potential overlap between benign and premalignant features.

Beyond increased lesion burden, immunosuppressed patients may also present with morphologically complex lesions, including hyperpigmented and leukoplakic plaques, which may correspond to high-grade disease. As shown in Figure 5, such features illustrate the clinical expression of vulvar HSIL in the setting of immunosuppression, emphasizing the importance of careful clinical assessment and a low threshold for biopsy.

### 5.4. Anal Canal and Perianal Region

HPV infection of the anal canal shares biological and histopathological features with cervical disease. Anal intraepithelial neoplasia (AIN) represents the precursor lesion to anal cancer and is strongly associated with high-risk HPV, particularly HPV16 [54].

Clinical presentations may range from asymptomatic lesions to symptoms such as pruritus, pain, or bleeding [54]. However, many cases remain undiagnosed without targeted evaluation. High-resolution anoscopy with directed biopsy is the gold standard for diagnosis in high-risk populations.

**Figure 6:**
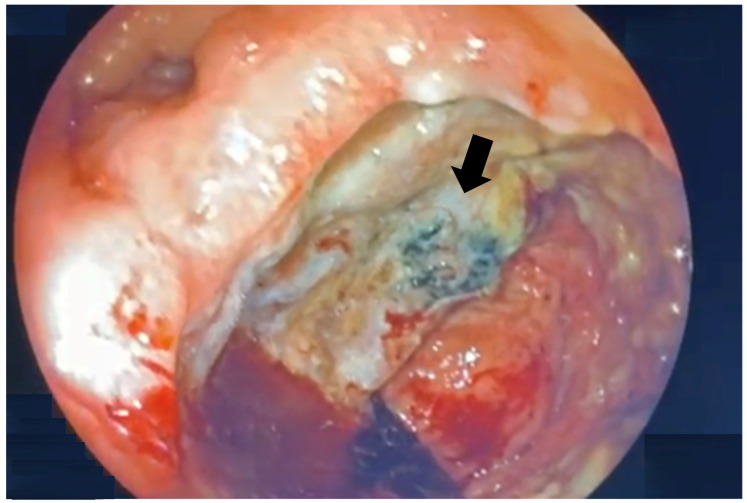
Anal squamous cell carcinoma in an immunosuppressed patient. Endoscopic view demonstrating invasive squamous cell carcinoma of the anal canal (black arrow) in a 55-year-old woman living with HIV, illustrating the malignant endpoint of HPV-related disease in the setting of immune dysfunction.

The presence of cervical or vulvar HSIL significantly increases the likelihood of anal involvement, reinforcing the concept of field cancerization and the need for expanded evaluation in selected patients. Thus, women with HPV-related genital intraepithelial neoplasia have a higher risk of anal intraepithelial lesions, reflecting the multicentric nature of anogenital infection. This pattern involves the cervix, vulva, and anal/perianal canal, supporting a field effect and the biological interconnection among these sites [55]. As illustrated in Figure 6, advanced disease may manifest as invasive squamous cell carcinoma of the anal canal, highlighting the potential for progression along the HPV-related disease spectrum, particularly in the setting of impaired immune competence.

### 5.5. Multifocal Disease and Field Effect

A defining feature of HPV infection is its propensity for multifocal involvement of the anogenital epithelium. The so-called “field effect” reflects the widespread exposure of epithelial surfaces to HPV and the ability of the virus to establish infection at multiple sites simultaneously or sequentially [50]. Rather than representing isolated lesions, HPV-related disease should be understood as a diffuse epithelial process in which genetically and virally altered fields predispose to the development of synchronous or metachronous lesions [54].

Multifocal disease is particularly prevalent in immunosuppressed populations, in whom impaired immune surveillance facilitates broader viral dissemination and persistence. This pattern is associated with increased diagnostic complexity, higher recurrence rates, and a greater risk of malignancy progression [51].

**Figure 7:**
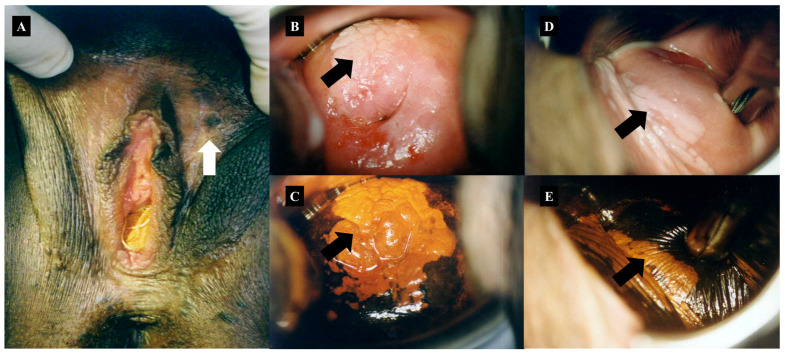
Multifocal HPV-related disease illustrating the field effect. Findings illustrate synchronous vulvar and cervical HSIL in a 30-year-old woman living with HIV, highlighting the multifocal nature of HPV infection in the setting of immune dysfunction. (**A**) Hyperpigmented vulvar plaques and papules (white arrow). (**B**) Cervix after acetic acid application showing dense acetowhite epithelium (black arrow). (**C**) Cervix following the Schiller test demonstrating central iodine-negative epithelium (black arrow; Schiller-positive). (**D**) Vagina after acetic acid application showing dense acetowhite epithelium (black arrow). (**E**) Vagina following the Schiller test demonstrating iodine-negative epithelium (black arrow).

As illustrated in Figure 7, simultaneous involvement of the vulva and cervix demonstrates the clinical expression of this field effect, with coexisting high-grade lesions across different anatomical sites. The presence of hyperpigmented vulvar plaques together with cervical abnormalities, such as leukoplakia, dense acetowhite epithelium, and iodine-negative areas on the Schiller test, highlights the importance of comprehensive evaluation of the entire lower genital tract. These findings reinforce the need for a systematic, site-spanning diagnostic approach and support the concept that HPV-related disease should be managed within an integrated anatomical and biological framework.

### 5.6. Clinical Implications of Site-Specific Distribution

The anatomical distribution of HPV-related lesions has direct implications for clinical practice. Identification of disease at one site should prompt careful evaluation of the entire lower genital tract, including the cervix, vagina, vulva, and, when appropriate, the anal region [24]. This comprehensive approach is essential for accurate diagnosis, appropriate risk stratification, and effective management [50].

Furthermore, site-specific differences in lesion biology and response to treatment underscore the importance of individualized care [48]. Immunosuppressed patients require heightened vigilance due to the increased likelihood of multifocal disease, persistence, and recurrence, reinforcing the need for tailored diagnostic and therapeutic strategies. Viewed as a whole, the anatomical distribution of HPV-related lesions reinforces the concept of a field effect, particularly in individuals living with immunosuppression, in whom impaired immune surveillance allows broader epithelial involvement and more complex clinical presentations.

The clinical characteristics and management strategies of HPV-related lesions across different anatomical sites are systematically compared in Table 1, highlighting site-specific patterns and the impact of immune status on clinical behavior and management.

## 6. Colposcopic and Vulvoscopic Evaluation

Colposcopy and vulvoscopy are complementary diagnostic tools in the evaluation of HPV-related disease, enabling detailed assessment of epithelial and vascular changes across the lower genital tract [48]. While colposcopy is well standardized and focused on the cervix and vagina, it should be interpreted within a risk-based model that integrates HPV status and cytology, with directed biopsies to improve diagnostic accuracy [56]. Vulvoscopy provides a magnified evaluation of vulvar lesions, albeit with greater reliance on clinical expertise due to lower standardization [56,57]. Together, these approaches are essential for identifying multifocal disease, guiding biopsies, and supporting accurate diagnosis [24].

### 6.1. Colposcopic Evaluation

Colposcopy remains a cornerstone in the diagnostic evaluation of HPV-related disease, providing a magnified, real-time assessment of epithelial and vascular changes in the cervix, vagina, and vulva. Its primary role is to identify areas suspicious for clinically significant disease and to guide targeted biopsies, thereby bridging screening findings and histopathological diagnosis [48]. Despite advances in molecular testing, colposcopy continues to be essential for lesion characterization and clinical decision-making, particularly in cases with discordant results or persistent HPV infection.

Colposcopic findings reflect morphofunctional epithelial alterations induced by HPV infection, with acetowhitening representing the principal visual marker of epithelial transformation, resulting from the coagulation of nuclear proteins following the application of acetic acid [48]. Low-grade lesions are typically characterized by thin acetowhite epithelium with indistinct margins and delicate vascular patterns, often associated with fine mosaicism and punctation, reflecting productive infection with lower oncogenic potential [57].

In contrast, high-grade lesions display dense, opaque acetowhite epithelium with rapid onset, well-defined and often raised borders, and abnormal vascular patterns such as coarse mosaicism, coarse punctation, and atypical vessels, reflecting architectural disorganization and tumor-associated angiogenesis [56]. Additional features, including the inner border sign and ridge sign, have high predictive value for HSIL and represent important markers in colposcopic assessment [48].

Despite these established criteria, the diagnostic accuracy of colposcopy remains moderate, with agreement between colposcopic impression and histopathology ranging from 59% to 65%, sensitivity for HSIL between 56% and 72%, and high specificity, often exceeding 90% [56]. These limitations underscore the need for directed biopsies whenever clinical suspicion exists, regardless of the apparent severity of colposcopic findings [24].

In this context, colposcopy evolves from a purely diagnostic tool into a critical component of risk-based clinical decision-making, integrating morphological assessment with individual patient risk, particularly in those with increased biological vulnerability, such as immunosuppressed populations, in whom lesions may be subtle, multifocal, or rapidly progressive.

### 6.2. Terminology and Standardization

Modern colposcopic practice is guided by the terminology proposed by the IFCPC, which established a standardized framework for describing findings and improving interobserver reproducibility [48]. This system emphasizes the evaluation of the squamocolumnar junction and transformation zone, replacing older concepts such as “satisfactory” and “unsatisfactory” examinations with a more objective and clinically meaningful classification [56,57].

Colposcopic findings are categorized into normal, grade 1 (minor), grade 2 (major), and suspicious for invasion [48]. This classification correlates with the likelihood of underlying histopathological abnormalities, with grade 1 findings generally associated with low-grade lesions and grade 2 findings more strongly predictive of HSIL [56]. The adoption of standardized terminology enhances communication, facilitates research comparability, and supports risk-based clinical management [24].

In contrast, vulvoscopy lacks a universally accepted classification system. Although general colposcopic principles can be applied, the interpretation of vulvar findings depends more heavily on morphological assessment and clinical judgment, reflecting the anatomical and histological heterogeneity of vulvar tissue.

### 6.3. Colposcopic Findings and Correlation with Disease Severity

Colposcopic interpretation is based on morphological changes induced by HPV infection. The most characteristic feature is acetowhitening following the application of acetic acid, which reflects increased nuclear density and protein coagulation within dysplastic cells [48].

Minor changes typically include thin acetowhite epithelium, indistinct borders, and fine vascular patterns such as fine punctation and fine mosaicism. These findings are commonly associated with low-grade lesions and productive infection [57]. In contrast, major changes, such as dense, opaque acetowhite epithelium with sharp borders, coarse punctation, coarse mosaicism, and abnormal vascular patterns, are more strongly associated with HSIL and carry a higher risk of clinically significant disease [56].

Specific features, including the inner border sign and ridge sign, have been described as having high predictive value for HSIL. However, colposcopy has inherent limitations, with only moderate diagnostic accuracy and notable interobserver variability. Agreement between colposcopic impression and histopathology ranges from 59% to 65%, with sensitivity for HSIL between 56% and 72% and high specificity, often exceeding 90% [56]. These limitations underscore the need for directed biopsies whenever clinical suspicion exists, regardless of the apparent severity of colposcopic findings [24].

Colposcopic findings and their correlation with the risk of high-grade disease are summarized in Table 2.

### 6.4. Technique and Systematic Examination

A high-quality colposcopic examination requires a systematic approach. The evaluation begins with inspection of the vulva, followed by examination of the vagina and cervix under increasing magnification [24,48]. Application of acetic acid (3–5%) is essential to highlight acetowhite changes, while the use of a green filter enhances visualization of vascular patterns, which are critical for identifying high-grade lesions [56,57].

The application of iodine solution (Schiller test) may assist in delineating abnormal epithelium, although its specificity is lower than that of acetic acid-induced changes. Accurate documentation, including lesion location, size, morphology, and visibility of the transformation zone, is fundamental for clinical follow-up and therapeutic planning. When available, photographic documentation improves longitudinal assessment and reproducibility [24,48].

### 6.5. Colposcopy in Immunosuppressed Populations

In immunosuppressed patients, colposcopic evaluation is often more challenging and clinically demanding. These individuals exhibit higher rates of persistent HPV infection, increased viral load, and a greater likelihood of multifocal disease [56]. Lesions may be more extensive, less well-defined, and more frequently involve multiple anatomical sites simultaneously [24].

As a result, colposcopy in this setting requires a broader and more meticulous examination, often with a lower threshold for biopsy. Multiple directed biopsies are frequently necessary to avoid underdiagnosis, particularly in the presence of subtle or heterogeneous lesions. In addition, persistent HPV infection, especially when type-specific, has been identified as a major predictor of recurrence, reinforcing the need for closer surveillance and shorter follow-up intervals in these populations [37,58].

## 7. Vulvar Examination Semiology and Criteria for Suspicion

Vulvar examination semiology is based on the integrated assessment of lesion characteristics, including morphology, color, surface texture, and vascular pattern, and is essential for the identification of clinically suspicious areas [48]. Lesions associated with vulvar HSIL frequently present as papules or plaques with heterogeneous morphological patterns, which may complicate clinical recognition when considered in isolation [59].

Lesion color may vary widely, ranging from whitish and erythematous to pigmented areas, without a consistent correlation with the degree of dysplasia, underscoring the need for contextual and integrative interpretation. Additional features such as hyperkeratosis, ulceration, nodularity, or architectural distortion should be regarded as warning signs suggestive of possible invasion and warrant prompt histopathological evaluation [59].

The decision to perform a biopsy should be guided by the presence of suspicious features, rapid lesion growth, or significant structural changes. In this context, biopsy plays a central role in early diagnosis and in reducing the risk of progression to invasive carcinoma, reinforcing its importance as a key component of vulvar assessment [48].

### 7.1. Vulvoscopy: Role and Limitations

Vulvar assessment, often referred to as vulvoscopy, complements colposcopy but lacks the same degree of standardization [48]. The anatomical complexity and variability of vulvar lesions limit the direct application of cervical colposcopic criteria [59]. Evaluation relies on careful inspection of surface morphology, including color, texture, thickness, and vascular patterns [48].

The use of acetic acid in the vulva is less specific and may cause discomfort, particularly in keratinized epithelium [48]. Therefore, its use should be selective. Given the relatively low diagnostic accuracy of visual assessment alone, biopsy remains essential for the definitive diagnosis of suspicious vulvar lesions. The diagnostic performance of vulvoscopy based solely on clinical criteria is limited, with agreement with histopathology reported to be around 50% [60]. Accordingly, vulvoscopy should be interpreted as a triage and lesion-localization tool rather than a definitive diagnostic method, reinforcing the need for histopathological confirmation whenever clinically indicated.

### 7.2. Integration of Colposcopy in Clinical Practice

The integration of colposcopy into clinical practice requires a comprehensive and site-inclusive approach. Given the high frequency of multifocal HPV-related disease, evaluation should extend beyond the cervix to include the vagina and vulva, and, in selected cases, the anal region [24,56]. Identification of lesions in one site should prompt careful assessment of others.

Colposcopy should not be viewed as an isolated diagnostic tool but as part of a broader diagnostic framework that includes cytology, HPV testing, and histopathology [48]. Its value lies in its ability to refine risk assessment, guide biopsies, and support individualized clinical decision-making [37].

In summary, colposcopy remains an indispensable tool in the management of HPV-related disease. Its effectiveness depends on standardized interpretation, technical proficiency, and integration with other diagnostic modalities, particularly in populations at increased risk, such as immunosuppressed women.

## 8. Screening, Triage, and Diagnosis

Cervical cancer prevention has undergone a major paradigm shift in recent years, moving from cytology-based screening toward molecular, risk-based strategies centered on HPV detection. This transition reflects the central role of persistent high-risk HPV infection in carcinogenesis and the superior sensitivity of HPV-based testing for identifying clinically significant disease. As a result, screening, triage, and diagnostic pathways have become increasingly integrated, with a focus on individualized risk stratification rather than rigid algorithmic approaches.

### 8.1. Screening in Immunocompetent Populations

In immunocompetent individuals, primary HPV testing has emerged as the preferred screening modality in many international guidelines. Detection of high-risk HPV (hrHPV) DNA provides superior sensitivity for identifying HSIL and precancerous disease, often exceeding 90%, compared with cytology alone, whose sensitivity typically ranges from 50% to 70% [24]. This increased diagnostic performance supports longer screening intervals following negative results and has driven a paradigm shift in cervical cancer prevention strategies.

Current recommendations vary slightly across organizations but converge on key principles. The American Cancer Society (ACS) recommends initiating screening at age 25 with primary HPV testing, whereas the U.S. Preventive Services Task Force (USPSTF) still considers cytology acceptable between 21 and 29 years, reflecting a transitional phase in guideline adoption [61]. The WHO, in turn, prioritizes HPV-based screening for women aged 30 to 49 years, with intervals of five to ten years, particularly in public health settings and low-resource environments [62]. This variability reflects differences in healthcare infrastructure and access to diagnostic technologies.

The incorporation of self-collected vaginal samples for hrHPV testing represents a significant advance in expanding access to screening. Recently approved by the U.S. Food and Drug Administration (FDA) and incorporated into U.S. guidelines, this approach demonstrates high concordance with clinician-collected samples, approaching 89%, and has the potential to reduce disparities in underserved populations [63,64].

### 8.2. Risk-Based Triage Strategies

The management of abnormal screening results has evolved toward a risk-based framework, largely driven by data-informed models that estimate the probability of underlying cervical intraepithelial neoplasia grade 3 or higher (CIN 3+). These risk-based models incorporate variables such as HPV status and genotype, cytology results, prior screening history, persistence of infection, previous histopathological findings, and prior treatment history to estimate the probability of CIN 3+. Contemporary recommendations from the ASCCP have replaced rigid algorithms with dynamic, individualized risk assessment based on current and prior screening results [24,65].

These models define clinical action thresholds according to estimated risk. For example, individuals with a CIN 3+ risk below 0.15% may safely return to routine screening at five-year intervals, whereas those with risks equal to or exceeding 25% warrant immediate intervention, including expedited colposcopy or excisional treatment [24]. Triage strategies following a positive HPV test may include cytology, HPV genotyping, or the use of molecular biomarkers.

Identification of high-risk genotypes, particularly HPV16 and HPV18, is associated with a substantially increased risk of HSIL and typically warrants immediate colposcopic evaluation, even in the absence of cytological abnormalities. Conversely, individuals with non-16/18 genotypes and normal cytology may be safely followed with repeat testing at defined intervals.

This shift toward dynamic risk estimation allows for more precise and individualized clinical management, reducing unnecessary procedures while ensuring timely identification of clinically significant disease and aligning screening strategies with underlying disease biology.

### 8.3. Screening in Immunosuppressed Populations

Screening strategies in immunosuppressed individuals differ substantially due to the higher prevalence of persistent infection, increased risk of progression, and reduced predictability of disease course. These populations, including individuals living with HIV, transplant recipients, and those receiving long-term immunosuppressive therapy, require more intensive and prolonged surveillance [66,67].

Screening is generally initiated earlier and performed at shorter intervals, often annually in the initial phases. However, screening strategies in individuals living with HIV vary across international guidelines, reflecting differing interpretations of the available evidence. U.S.-based recommendations, including those from the NIH Office of AIDS Research and the HIV Medicine Association/Infectious Diseases Society of America (HIVMA/IDSA), continue to prioritize cytology as the primary screening modality, recommending annual testing, with the possibility of extending intervals to three years after consecutive normal results [21].

In contrast, the WHO advocates for primary HPV DNA testing within a “screen–triage–treat” framework, initiating screening at age 25 and recommending intervals of three to five years, emphasizing the superior sensitivity of HPV-based approaches [66]. This divergence highlights ongoing transitions in screening paradigms for this high-risk population.

The high prevalence of HPV infection in women living with HIV further complicates screening strategies, with up to 16% demonstrating abnormal cytology on serial evaluations, including atypical squamous cells of undetermined significance (ASC-US) or more severe abnormalities. In this context, HPV-based screening strategies have been proposed to optimize detection while reducing unnecessary procedures, with evidence suggesting up to a 50% reduction in unnecessary colposcopies compared with co-testing approaches [66].

Another important consideration is the lack of recommendation for discontinuation of screening after 65 years of age in this population, in contrast to the general population, due to the persistent risk of neoplastic progression [21]. Furthermore, expanded evaluation of the lower anogenital tract, including the anal canal and vulva, should be considered, given the field effect associated with HPV infection [67].

**Figure 8:**
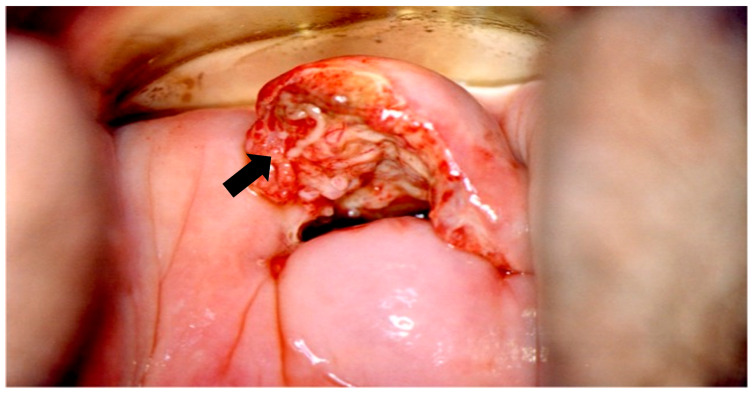
Early-onset cervical squamous cell carcinoma in an immunosuppressed patient. Speculum examination demonstrating invasive squamous cell carcinoma of the cervix (black arrow) in a 19-year-old woman living with HIV, illustrating the potential for accelerated progression of HPV-related disease in the setting of immune dysfunction.

This intensified approach reflects the increased risk of persistent infection, rapid progression, and earlier onset of high-grade lesions and invasive disease in immunosuppressed individuals. As illustrated in Figure 8, invasive cervical carcinoma may occur at a young age in patients with impaired immune function, underscoring the need for early detection strategies and vigilant surveillance in this high-risk population.

Non-HIV-related immunosuppression comprises a heterogeneous group of clinical conditions, including solid organ and hematopoietic stem cell transplant recipients, patients with autoimmune diseases, and individuals receiving long-term immunosuppressive or biologic therapies, all of whom may have an increased risk of cervical neoplasia [61]. Although the absolute risk varies across conditions, there is broad consensus that immunosuppression impairs HPV clearance, promoting viral persistence and increasing the likelihood of progression to precursor lesions [68].

Screening recommendations for these populations are largely based on expert consensus, reflecting the limited availability of high-quality prospective data. Guidelines from the NCCN suggest considering more frequent and intensive screening strategies, particularly in high-risk groups such as hematopoietic stem cell transplant recipients [69]. In addition, the presence of immunosuppression is generally regarded as a criterion for continuing screening beyond the age of 65, irrespective of prior screening history, due to the sustained risk of neoplastic progression [24].

Despite these recommendations, recent systematic reviews highlight substantial gaps in the standardization of screening strategies in this population, including uncertainties regarding the optimal age of initiation, screening intervals, and preferred testing modalities [68]. This heterogeneity underscores the need for dedicated studies to better define risk-adapted screening approaches and to support the development of more robust and individualized guidelines.

Unlike the general population, discontinuation of screening based on age alone is not recommended, given the persistently elevated risk of HPV-related disease. Although cytology has traditionally been the cornerstone of screening in these populations, there is increasing support for the incorporation of HPV DNA testing as a primary or adjunctive tool, given its higher sensitivity.

Importantly, the high prevalence of HPV infection in patients with altered immune competence poses challenges for specificity, leading to a greater number of positive tests and potential over-referral. Therefore, careful triage and judicious use of colposcopy are essential to balance early detection with avoidance of unnecessary interventions.

### 8.4. Diagnostic Modalities

Definitive diagnosis of HPV-related lesions relies on histopathological evaluation obtained through directed biopsy. Colposcopy serves as the central diagnostic tool guiding biopsy, allowing targeted sampling of suspicious areas. However, its diagnostic accuracy is inherently limited, reinforcing the importance of combining morphological assessment with molecular testing.

Advances in molecular biology have enabled the development of more sensitive and specific diagnostic methods for the detection of cervical precancerous lesions. Among these, HPV DNA and HPV messenger ribonucleic acid (mRNA) assays are widely used in screening programs [70,71]. While HPV DNA testing identifies the presence of viral genetic material, mRNA-based assays detect the expression of oncogenic proteins E6 and E7, which are directly associated with transforming viral activity.

Comparative studies demonstrate that both methods have similar sensitivity for the detection of CIN 2+ and CIN 3+, typically ranging from 92% to 95%, whereas mRNA assays offer slightly higher specificity, reducing false-positive results and potentially decreasing unnecessary diagnostic procedures [70,72]. This distinction may have meaningful clinical implications, particularly in optimizing triage strategies.

The role of HPV genotyping has also expanded, enabling the identification of high-risk genotypes such as HPV16 and HPV18, whose presence warrants immediate colposcopic evaluation regardless of cytological findings [73]. More recently, extended genotyping panels incorporated into ASCCP recommendations have allowed for more refined risk stratification, supporting increasingly individualized clinical decision-making.

The role of epigenetic biomarkers has also gained increasing attention in recent years, particularly DNA methylation assays involving both host and viral genes. Aberrant methylation patterns are closely associated with persistent transforming HPV infection, viral integration, and progression to high-grade lesions and invasive carcinoma. In this context, methylation assays may contribute to improved risk stratification by distinguishing transient infections from clinically significant transforming disease, especially among HPV-positive individuals with equivocal cytology or persistent infection. Emerging evidence suggests that these assays may complement HPV-based screening and biomarker strategies, potentially reducing unnecessary colposcopic referrals while improving identification of patients at higher oncogenic risk. Nevertheless, their incorporation into routine clinical practice remains heterogeneous, and further validation is still required, particularly in immunosuppressed populations.

The use of biomarkers has further enhanced diagnostic accuracy, particularly with the dual-stain p16/Ki-67 assay, which detects concurrent expression of p16, a marker of oncogenic activity, and Ki-67, a marker of cellular proliferation, reflecting cell cycle deregulation [74,75]. This approach demonstrates high sensitivity for the detection of high-grade lesions and improved specificity compared with conventional cytology, significantly reducing unnecessary colposcopic referrals [1,76]. In addition, dual-stain testing enables more accurate risk stratification, as individuals with positive HPV tests but negative p16/Ki-67 results have a low risk of progression, allowing for extended follow-up intervals [74].

Despite these advances, important limitations remain. The performance of molecular and biomarker-based assays may be reduced in self-collected samples and in immunosuppressed populations, where altered immune responses and viral dynamics may affect test accuracy [77].

### 8.5. Special Considerations: Multifocal Disease and Expanded Evaluation

A critical aspect of HPV-related disease is its tendency toward multifocal involvement of the anogenital tract. The presence of cervical HSIL increases the likelihood of synchronous lesions in the vagina, vulva, and anal canal. This pattern is particularly pronounced in immunosuppressed individuals, in whom viral persistence and dissemination are more common.

Accordingly, diagnostic evaluation should not be limited to the cervix. Careful inspection of the vagina and vulva is essential during colposcopic examination, and targeted assessment of the anal region may be indicated in selected high-risk populations. Anal cytology and high-resolution anoscopy may play a role in this expanded diagnostic approach, particularly in individuals with known lower genital tract disease.

### 8.6. Integration into Clinical Practice

Screening, triage, and diagnostic strategies for HPV-related disease must be viewed as components of a continuous and integrated care pathway. The transition from population-based screening to individualized risk assessment represents a major advance, enabling more precise and effective clinical management.

In immunocompetent populations, this approach allows safe extension of screening intervals while maintaining high sensitivity for clinically significant disease. In contrast, immunosuppressed individuals require intensified surveillance, broader anatomical assessment, and a lower threshold for diagnostic intervention.

Ultimately, the effectiveness of screening and diagnostic strategies depends on their integration into organized healthcare systems, equitable access to appropriate tools, and adherence to evidence-based protocols. Tailoring these approaches according to immunological status is essential to optimize outcomes and reduce the global burden of HPV-related malignancies. This transition toward risk-based screening represents one of the most significant advances in cervical cancer prevention, enabling a more precise alignment between disease biology and clinical management.

## 9. Vaccination

Prophylactic vaccination against HPV represents a cornerstone of primary prevention, with a profound impact on the incidence of persistent infection, HSIL, and HPV-related malignancies. By targeting the most oncogenic genotypes, vaccination interrupts the earliest stages of carcinogenesis and complements screening-based strategies. Its integration into global health policies has been central to efforts aimed at eliminating cervical cancer as a public health problem [78].

### 9.1. Global Recommendations and Dosing Strategies

Recent recommendations from the WHO, through the Strategic Advisory Group of Experts on Immunization (SAGE), have introduced a paradigm shift in HPV vaccination strategies, particularly with the endorsement of simplified dosing schedules for immunocompetent individuals. A single-dose regimen has been proposed as an effective alternative for girls aged 9–14 years and young women up to 20 years, based on robust evidence demonstrating sustained immunogenicity and long-term protection comparable to multi-dose schedules [79,80]. Randomized trials, including the IARC India Trial and the Costa Rica Vaccine Trial, have demonstrated persistent antibody levels for over a decade, supporting the durability of simplified regimens [79,80].

This approach is biologically supported by the stronger immune response observed in younger individuals, in whom antibody titers are often equivalent or superior to those achieved in older populations receiving three-dose schedules. Accordingly, one- or two-dose schedules are now widely accepted for immunocompetent individuals, depending on age and local policy frameworks, with a minimum interval of five months required between two doses to ensure adequate immunogenicity [79,81].

In contrast, immunosuppressed populations, including individuals living with HIV, transplant recipients, and those receiving immunosuppressive or biologic therapies, require a three-dose schedule. This recommendation reflects concerns regarding attenuated immune responses and the need to ensure adequate and sustained protection in the context of impaired host immunity [21].

### 9.2. Efficacy and Population Impact in Immunocompetent Individuals

HPV vaccines demonstrate exceptionally high prophylactic efficacy, particularly when administered prior to viral exposure. Clinical trials have shown efficacy exceeding 99% in preventing persistent infection and precursor lesions associated with vaccine-covered genotypes, especially HPV16 and HPV18 [79,82]. The nonavalent vaccine further expands protection by including additional oncogenic types, consolidating its role in primary prevention [79].

Population-level data confirm the effectiveness of vaccination programs, with reductions of up to 88% in vaccine-type HPV prevalence among adolescents following the introduction of the quadrivalent vaccine, along with significant declines in older age groups [83]. Notably, indirect protection has also been observed in unvaccinated individuals, reflecting herd immunity effects [83,84].

Long-term studies demonstrate sustained protection for at least 10–12 years without evidence of waning immunity, supporting the absence of current recommendations for booster doses in immunocompetent individuals [79]. Although vaccination is most effective prior to sexual debut, benefits have also been observed in previously exposed individuals, including reductions in composite HPV-related outcomes, albeit with lower magnitude of effect [85]. Importantly, vaccines have no therapeutic effect on established infections, reinforcing their prophylactic nature [86].

### 9.3. Vaccination in Immunosuppressed Populations

In immunosuppressed individuals, HPV vaccination remains safe and immunogenic, although the magnitude of the immune response may be reduced compared with immunocompetent populations. In people living with HIV, seroconversion rates are typically high, often approaching 100%, but antibody titers may be lower, and immune response may correlate with CD4 count and viral suppression status [10].

Among solid organ transplant recipients, immunogenicity is significantly reduced, with seroconversion rates often below 70%, reflecting the impact of chronic immunosuppressive therapy on B- and T-cell responses [87]. Similarly, patients with autoimmune diseases receiving immunosuppressive or biologic therapies demonstrate adequate safety profiles but potentially attenuated immune responses [10,87].

Despite these limitations, vaccination is strongly recommended across these populations due to their increased risk of persistent HPV infection and HPV-related malignancies. Importantly, vaccination should ideally be administered before the onset of immunosuppression whenever possible [79].

### 9.4. Vaccination After HPV Exposure

Although HPV vaccines are prophylactic and do not treat established infections, evidence suggests that vaccination after exposure may confer partial benefit by protecting against non-acquired genotypes and potentially reducing recurrence after treatment of HSIL [85]. However, the magnitude of this effect is variable and depends on prior exposure and immunological status [86].

### 9.5. Integration with Screening and Public Health Strategies

Vaccination does not eliminate the need for screening, as current vaccines do not cover all oncogenic HPV types and have no effect on pre-existing infections. Approximately 10% of cervical cancer cases are attributed to genotypes not included in current vaccines [79].

Mathematical models suggest that screening strategies may be modified in highly vaccinated populations, with later initiation and longer intervals, while maintaining cost-effectiveness [88,89]. However, these approaches remain prospective and have not yet been incorporated into current clinical guidelines. Thus, vaccination and screening should be viewed as complementary and synergistic strategies, essential for sustained reduction in HPV-related disease burden [24].

### 9.6. Future Directions and Unmet Needs

Despite substantial advances in HPV prevention and control, important gaps remain, particularly in populations with altered immune competence. One of the major challenges lies in optimizing vaccination strategies for immunosuppressed individuals, in whom immunogenicity may be attenuated and the duration of protection remains incompletely defined. The role of booster doses in these populations is still uncertain and warrants further investigation.

In addition, significant limitations persist in the evidence base guiding screening and management in immunosuppressed groups. Current recommendations are largely extrapolated from studies conducted in immunocompetent populations, underscoring the need for prospective, population-specific research to define optimal screening intervals, diagnostic pathways, and therapeutic strategies.

The development and validation of novel biomarkers, as well as the integration of molecular diagnostics into risk-based algorithms, represent promising avenues to improve precision in clinical decision-making. Similarly, advances in therapeutic vaccines may offer future strategies for the treatment of established HPV-related disease, potentially addressing a critical unmet need beyond prophylaxis.

From a public health perspective, expanding vaccination coverage remains a central priority, particularly in low- and middle-income countries, where the burden of HPV-related disease is highest. Achieving global elimination targets will depend not only on vaccination uptake but also on the effective integration of vaccination, screening, and treatment within equitable healthcare systems.

Ultimately, the future of HPV prevention lies in the transition from uniform strategies to individualized, risk-adapted approaches that account for immunological status, viral factors, and population-specific vulnerabilities. In this context, bridging current knowledge gaps will be essential to refine clinical guidelines and to ensure that the benefits of prevention are equitably distributed across all populations.

## 10. Principles of Treatment and Clinical Decision-Making

The treatment of HPV-related disease in the female lower genital tract is fundamentally guided by lesion grade, anatomical site, and host immunological status. Contemporary strategies are increasingly structured around risk-based models, in which the probability of progression to HSIL or invasive carcinoma determines the need for intervention. While conservative management remains appropriate for low-grade lesions with a high likelihood of spontaneous regression, HSIL requires active treatment, and immunosuppressed patients demand a more vigilant and often more aggressive approach due to higher rates of persistence, recurrence, and multifocal disease.

### 10.1. Treatment of Vulvar Disease

The management of vulvar HPV-related disease requires a careful balance between therapeutic efficacy and preservation of anatomical integrity and function. The diagnosis of anogenital warts is clinical, and HPV testing is not recommended in this context, as it does not influence management [9]. Biopsy is reserved for atypical lesions, such as those that are pigmented, indurated, ulcerated, or fixed to deeper planes, or in cases of treatment failure, particularly in patients with impaired immunity [9,90,91]. From a histopathological standpoint, these lesions typically demonstrate diffuse overexpression of p16 along with a wild-type p53 immunostaining pattern [92]. There is no universally superior treatment modality, and therapeutic decisions must be individualized according to lesion characteristics, patient preference, and clinical context [9].

Patient-applied therapies include imiquimod, administered either as a 5% formulation three times weekly for up to 16 weeks or as a 3.75% formulation applied daily for up to 8 weeks, as well as podofilox 0.5%, applied twice daily for three consecutive days followed by four days off in repeated cycles [93]. Sinecatechins 15% may also be used, typically applied three times daily for up to 16 weeks, although their use should be avoided in immunosuppressed individuals due to the absence of safety data. In refractory cases, topical cidofovir at concentrations of 1–3% may be considered off-label, particularly in patients with compromised immune status.

**Figure 9:**
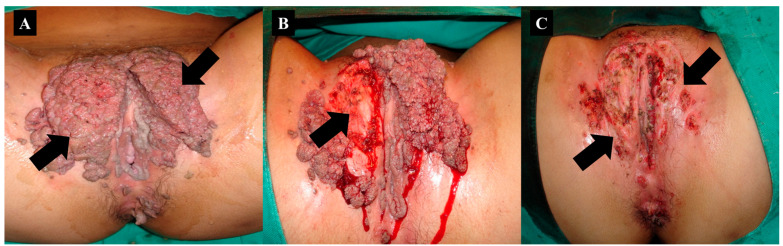
Extensive vulvar condylomatous disease requiring surgical management. (**A**) Giant vulvar condyloma acuminatum in a 16-year-old patient living with HIV, demonstrating extensive and confluent lesions (black arrows). (**B**) Surgical excision of the lesions using high-frequency electrosurgery (black arrow). (**C**) Postoperative view following lesion excision (black arrows), illustrating the role of operative treatment in cases of high lesion burden and refractory disease.

Provider-administered treatments include cryotherapy with liquid nitrogen, usually performed in freeze–thaw cycles of 10 to 20 s and repeated at one- to two-week intervals, and the application of trichloroacetic or bichloroacetic acid at concentrations of 80–90%, applied weekly. Surgical excision remains the preferred option for large, keratinized, refractory, or clinically suspicious lesions, allowing histopathological evaluation, while carbon dioxide laser ablation offers precise tissue destruction and is particularly useful in multifocal or anatomically complex disease. As illustrated in Figure 9, extensive vulvar condylomatous disease may require surgical excision, particularly in cases of high lesion burden and functional impairment, highlighting the role of operative management in selected patients.

In patients living with HIV, lesions are typically more extensive, associated with higher viral burden, and characterized by increased recurrence rates, often requiring combined therapeutic approaches. In this context, closer surveillance is essential, with a low threshold for biopsy and careful exclusion of HSIL. Recurrence remains a major challenge, particularly in immunocompromised patients, in whom lesions are more frequently multifocal and resistant to therapy.

**Figure 10:**
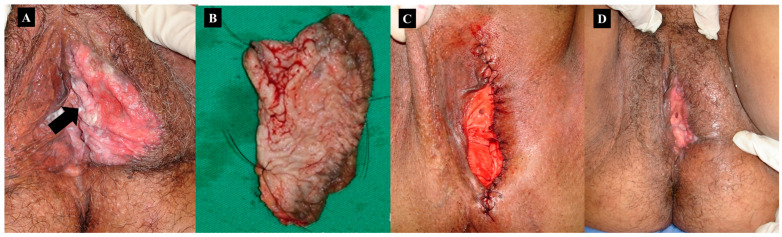
Surgical management of vulvar HSIL in an immunosuppressed patient. (**A**) Vulvar high-grade squamous intraepithelial lesion (HSIL, usual-type VIN) in a 58-year-old woman living with HIV (black arrow). (**B**) Surgical specimen following wide local excision. (**C**) Immediate postoperative view demonstrating vulvar reconstruction and wound closure. (**D**) Late postoperative view showing satisfactory healing and anatomical restoration.

Vulvar HSIL represents the classic HPV-related premalignant pathway and requires active treatment in all cases. Therapeutic options include surgical excision, laser ablation, and topical imiquimod, as recommended by the ACOG [93]. Evidence from the randomized PITVIN trial demonstrated that topical imiquimod is non-inferior to surgical treatment, with comparable short-term complete response rates [94]. Despite adequate treatment, recurrence rates remain high, reaching up to 50%, and are influenced by factors such as positive margins, multifocality, and delayed therapeutic response [95]. In this context, surgical excision plays a central role, particularly for extensive or clinically suspicious lesions, allowing both complete removal and histopathological assessment. The risk of progression from vulvar HSIL to invasive carcinoma is relatively low, estimated at approximately 4.5% within five years. As illustrated in Figure 10, wide local excision represents an effective approach for vulvar HSIL, encompassing lesion removal, specimen evaluation, and postoperative reconstruction, highlighting the importance of individualized surgical management in patients with complex disease.

**Figure 11:**
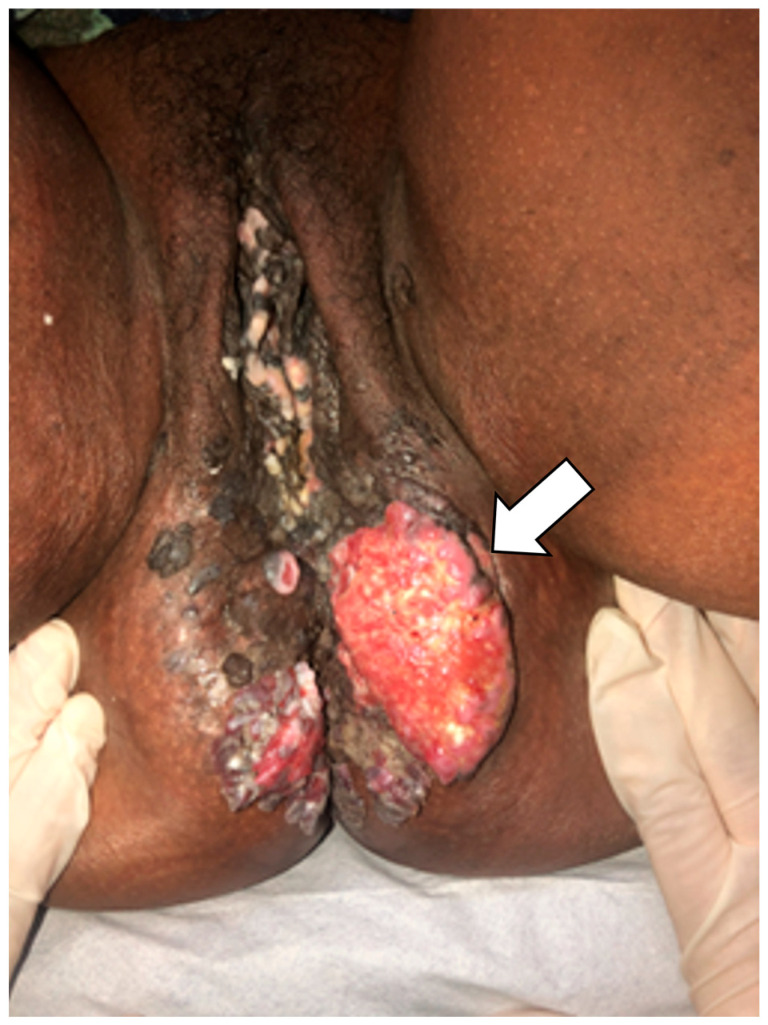
Vulvar squamous cell carcinoma associated with multifocal HSIL in an immunosuppressed patient. Extensive vulvar squamous cell carcinoma (white arrow) coexisting with vulvar and anal HSIL in a 55-year-old renal transplant recipient receiving immunosuppressive therapy, illustrating the coexistence of premalignant and invasive disease in the setting of chronic immune dysfunction.

In advanced or neglected cases, particularly in the setting of chronic immunosuppression, HSIL may coexist with or progress to invasive carcinoma. As illustrated in Figure 11, vulvar squamous cell carcinoma may arise in association with multifocal HSIL involving both vulvar and perianal regions, underscoring the aggressive behavior and diagnostic complexity of HPV-related disease in immunocompromised patients.

**Figure 12:**
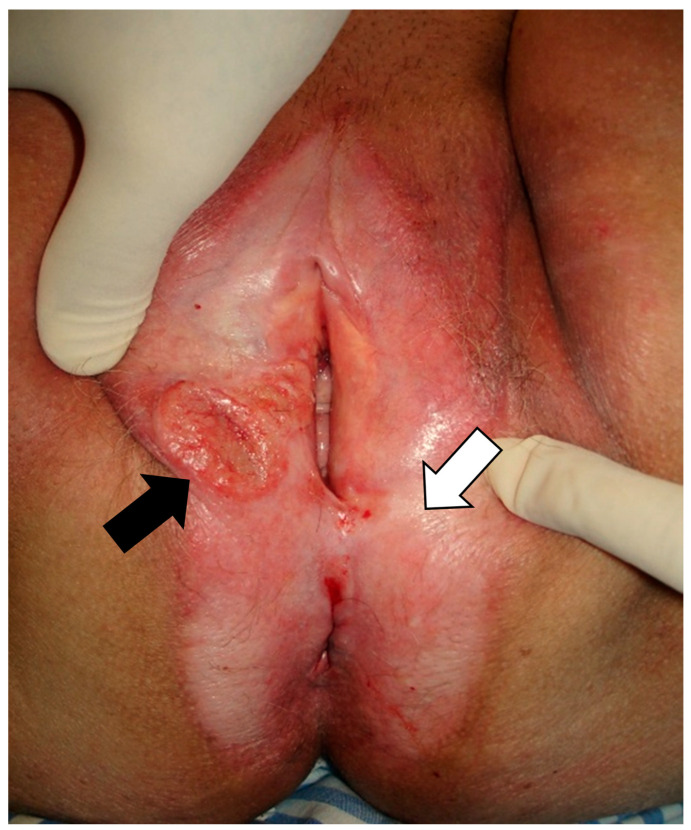
Vulvar squamous cell carcinoma arising in the setting of lichen sclerosus. Hypopigmented vulvar area with loss of labial architecture and clitoral burying, consistent with lichen sclerosus (white arrow), associated with an exophytic and ulcerated lesion on the inner aspect of the right labium majus extending to the vestibule, diagnosed as vulvar squamous cell carcinoma (black arrow), in a 65-year-old woman with chronic corticosteroid use for idiopathic thrombocytopenic purpura.

In contrast, differentiated vulvar intraepithelial neoplasia represents an HPV-independent pathway with markedly aggressive behavior, characterized by high rates of progression and short intervals to invasive carcinoma. Surgical excision with adequate margins is mandatory in these cases, and topical therapies are not indicated. The frequent association with lichen sclerosus requires concomitant treatment with high-potency topical corticosteroids, which reduces the risk of malignant transformation. As illustrated in Figure 12, vulvar carcinoma arising in the setting of chronic lichen sclerosus demonstrates the characteristic features of this pathway, including architectural distortion, hypopigmented areas, and destructive invasive lesions, highlighting the importance of early recognition and appropriate management of this high-risk condition.

### 10.2. Treatment of Vaginal Disease

The management of vaginal intraepithelial lesions is constrained by anatomical considerations and the need to preserve vaginal function. Low-grade lesions may be managed expectantly with careful surveillance, whereas HSIL requires active treatment. Carbon dioxide laser ablation is commonly used due to its ability to precisely target superficial lesions while preserving underlying structures. Topical 5-fluorouracil may be used in selected cases, typically administered intravaginally at weekly intervals over several weeks, although tolerability is often limited by local irritation. Surgical excision is reserved for localized or refractory lesions, particularly when invasion cannot be excluded. Recurrence is not uncommon and is significantly more frequent in immunosuppressed patients, necessitating prolonged follow-up and, in some cases, repeated interventions.

### 10.3. Treatment of Cervical Disease

The management of cervical high-grade squamous intraepithelial lesions (HSILs; cervical intraepithelial neoplasia [CIN] 2/3) has evolved substantially with the introduction of risk-based guidelines from the ASCCP, which incorporate individualized estimates of progression to CIN 3 or higher, replacing approaches based solely on isolated test results [24,96].

Treatment is recommended for all cases of CIN 3, except in specific circumstances such as pregnancy, due to the high risk of progression to invasive [24]. For CIN 2, treatment is also generally recommended; however, observation may be considered in carefully selected patients, particularly those with reproductive desires, provided that the squamocolumnar junction is fully visualized and there is no evidence of high-grade disease on endocervical sampling [73].

Observation of CIN 2 requires close surveillance with colposcopy and HPV-based testing at six-month intervals for up to two years, with treatment indicated in cases of persistence [96]. This approach is supported by the heterogeneous biological behavior of CIN 2, which demonstrates higher rates of spontaneous regression compared with CIN 3, especially in younger women [24].

Excisional procedures, such as loop electrosurgical excision procedure (LEEP) or cold-knife conization, are generally preferred, while ablative methods may be considered in selected cases [24]. Non-surgical therapies are not recommended outside of clinical trials, and hysterectomy should not be used as a primary treatment for HSIL [24].

Recurrence after treatment is not negligible, ranging from 5% to 25%, and is influenced by factors such as positive surgical margins, which may increase the risk of treatment failure by up to fivefold [97]. In this context, post-treatment HPV testing has emerged as the most accurate predictor of recurrence, with high sensitivity and a negative predictive value approaching 100% [97].

Follow-up includes HPV testing at six months, followed by annual surveillance until three consecutive negative results are obtained, and subsequently every three years for at least 25 years, regardless of patient age [24,97].

In immunosuppressed patients, management should be more aggressive, with referral to colposcopy for any cytological abnormality associated with HPV detection, a greater preference for excisional methods, and prolonged follow-up due to higher recurrence rates, which may reach 40–60% [24,97].

### 10.4. Anal Canal

The management of anal intraepithelial lesions follows similar principles but is guided by high-resolution anoscopy, which allows targeted identification and treatment of HSIL. Ablative therapies, including infrared coagulation, electrocautery, and laser ablation, are commonly employed, while topical agents such as imiquimod or 5-fluorouracil may be used in selected cases, particularly in multifocal disease. Excisional procedures are reserved for larger or suspicious lesions. Recurrence is particularly frequent in individuals living with human immunodeficiency virus, often requiring repeated treatments and long-term surveillance [40].

### 10.5. Follow-Up and Clinical Integration

Across all anatomical sites, recurrence remains a defining feature of HPV-related disease and is closely linked to viral persistence and host immune status. Follow-up strategies typically involve HPV testing, cytology, and site-specific examination. In immunocompetent individuals, surveillance intervals may be progressively extended following negative results, whereas immunosuppressed patients require more intensive and prolonged follow-up, often without a clearly defined endpoint.

Immunological status is a fundamental determinant of treatment strategy. In immunocompetent patients, management can often follow standard risk-based guidelines, with an emphasis on avoiding unnecessary interventions. In contrast, patients with impaired immunity require a more aggressive and individualized approach, characterized by earlier intervention, broader anatomical evaluation, and closer follow-up. This distinction reflects differences in disease biology, including higher viral load, increased likelihood of multifocal disease, and impaired immune-mediated clearance. As such, clinical decision-making must incorporate not only lesion characteristics but also the broader immunological context.

**Figure 13:**
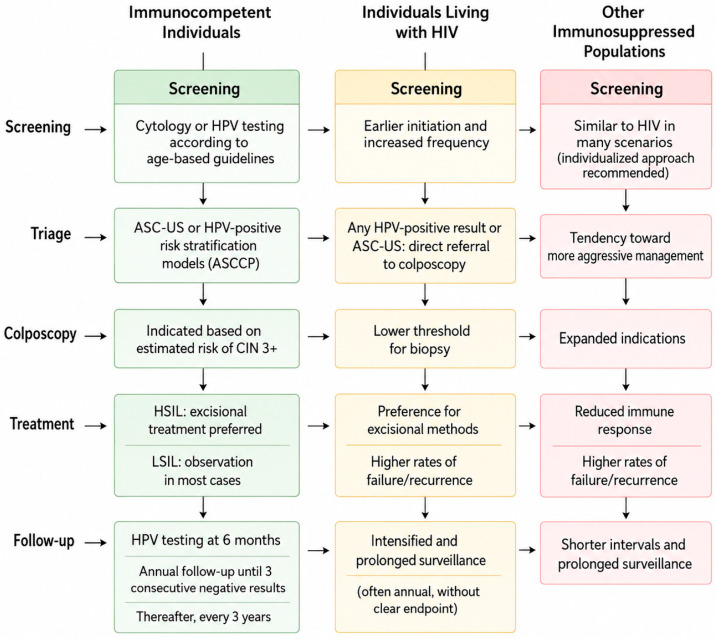
Risk-adapted approaches to screening, colposcopy, treatment, and follow-up of HPV-related disease across different immunological contexts. Immunosuppressed populations require intensified surveillance, lower thresholds for colposcopy and biopsy, and prolonged follow-up due to higher risks of persistence, recurrence, and progression. ASC-US: atypical squamous cells of undetermined significance. HPV: human papillomavirus. HIV: human immunodeficiency virus. HSIL: high-grade squamous intraepithelial lesion. LSIL: low-grade squamous intraepithelial lesion. CIN: cervical intraepithelial neoplasia. ASCCP: American Society for Colposcopy and Cervical Pathology.

The application of these strategies across different immunological contexts is schematically illustrated in Figure 13.

### 10.6. Integrated Perspective: Immunocompetent vs. Immunosuppressed HPV Infection

A comparative analysis of HPV infection in immunocompetent and immunosuppressed women consistently demonstrates the coexistence of shared biological principles and markedly divergent clinical trajectories. While the fundamental mechanisms of HPV-related carcinogenesis remain conserved, immunological status profoundly influences viral persistence, disease expression, and clinical outcomes, with direct implications for diagnosis, management, and public health strategies.

Across different clinical contexts, the core diagnostic framework remains unchanged. The evaluation of HPV-related disease continues to rely on the integration of cytological and/or molecular screening, colposcopic assessment, and histopathological confirmation. The requirement for tissue diagnosis in the presence of suspicious findings is a universal principle, supported by the limited diagnostic accuracy and interobserver variability of colposcopy, particularly in low-grade lesions [24,56]. In this setting, directed biopsy remains the diagnostic gold standard, especially in cases of discordant screening results or features suggestive of high-grade disease.

At a biological level, type-specific viral persistence remains the central determinant of progression to high-grade intraepithelial lesions and invasive carcinoma, regardless of immune status [70,79]. This concept underpins the central role of high-risk HPV testing in contemporary screening strategies, as well as the incorporation of molecular biomarkers, such as p16/Ki-67, to refine risk stratification [74]. Thus, although absolute risk varies between populations, the fundamental pathways of HPV-driven carcinogenesis are shared.

In contrast, immunosuppression significantly reshapes the clinical phenotype of HPV infection, imposing a pattern of increased aggressiveness and complexity. Immunosuppressed women exhibit higher rates of persistent infection, greater viral genotype diversity, and increased prevalence of multiple concurrent infections, translating into a higher burden of disease and an elevated risk of neoplastic progression [66]. This effect is particularly pronounced in women living with HIV, in whom the risk of cervical cancer may be up to sixfold higher than in the general population, even in the context of antiretroviral therapy [67].

Another defining feature in this population is the increased frequency of multifocal disease and the so-called field effect of HPV infection. The simultaneous involvement of the cervix, vagina, vulva, and anal canal is substantially more common, reflecting the impaired capacity of the immune system to control viral dissemination [51,54]. This pattern necessitates a broader and more systematic evaluation of the lower anogenital tract, including detailed vulvar inspection and, in selected cases, targeted anal assessment.

Recurrence following treatment further highlights the divergence between immunological contexts. While recurrence rates after HSIL treatment in immunocompetent women range from 5% to 25%, these rates may reach 40–60% in immunosuppressed individuals, reflecting persistent viral activity and impaired local immune response [97]. This observation supports the need for more intensive and prolonged follow-up strategies, with shorter surveillance intervals and a lower threshold for intervention.

These differences translate directly into distinct screening and follow-up strategies. International guidelines consistently recommend earlier initiation of screening, shorter intervals between evaluations, and the absence of an upper age limit for screening cessation in immunosuppressed populations [21,98]. Such adaptations reflect the sustained lifetime risk of disease progression in these individuals.

Despite advances in the understanding of HPV infection in immunosuppressed populations, significant gaps remain, particularly in non-HIV-related immunosuppression. In contrast to women living with HIV, for whom evidence is more robust and guidelines are relatively well established, data regarding transplant recipients, patients with autoimmune diseases, and those receiving immunobiologic therapies remain heterogeneous and largely derived from observational studies or expert consensus [61,68]. This heterogeneity limits the standardization of clinical recommendations, especially regarding the optimal age of screening initiation, screening intervals, and follow-up strategies.

From a public health perspective, these findings underscore the need for differentiated strategies tailored to high-risk populations. The global goal of cervical cancer elimination, as proposed by the WHO, is unlikely to be achieved without the effective inclusion of immunosuppressed individuals in intensified screening programs and equitable access to diagnosis and treatment [62,99]. This includes not only expanding vaccination coverage but also ensuring organized screening, adequate follow-up, and timely therapeutic interventions.

In addition, these data highlight the importance of specialized training in colposcopy and systematic vulvar evaluation. The increased frequency of multifocal lesions and the greater diagnostic complexity observed in immunosuppressed patients require clinicians skilled in comprehensive lower genital tract assessment. Standardized examination protocols and proper documentation are essential to improve diagnostic accuracy and longitudinal patient care [48,56].

Ultimately, the integration of vaccination, screening, and individualized clinical management emerges as the central strategy for reducing the burden of HPV-related disease. In immunosuppressed populations, this integration must be even more rigorous, given the higher likelihood of failure of isolated interventions. The transition toward risk-based, personalized approaches represents a major advance, enabling more precise and context-adapted clinical decision-making.

From a broader perspective, while the fundamental principles of HPV-related disease remain consistent across populations, immunosuppression defines a distinct clinical phenotype characterized by increased viral persistence, multifocality, recurrence, and risk of progression [100]. Recognizing and addressing these differences is essential for optimizing clinical care, refining public health strategies, and guiding future research agendas.

### 10.7. Future Directions and Emerging Approaches

Despite advances in surgical and ablative therapies, current treatments primarily address the consequences of HPV infection rather than the underlying viral persistence. This limitation has driven interest in novel approaches, including therapeutic vaccines and immunomodulatory strategies aimed at enhancing host immune responses.

While these approaches remain investigational, they hold promise for improving outcomes, particularly in patients with recurrent or refractory disease. Integration of such strategies into clinical practice may, in the future, shift the paradigm from lesion-directed treatment toward disease-modifying interventions.

Ultimately, the management of HPV-related disease requires a nuanced and individualized approach that integrates lesion characteristics, anatomical distribution, and host immune status. By aligning treatment strategies with underlying disease biology, clinicians can optimize outcomes while minimizing unnecessary interventions. This approach is particularly critical in immunosuppressed populations, in whom the burden of disease is greater and the margin for error is narrower.

## 11. Future Directions, Evidence Gaps, and Public Health Implications

Despite substantial advances in HPV-related disease prevention and management, important gaps in evidence remain, particularly regarding immunosuppressed populations beyond HIV infection. Most current recommendations are derived from studies involving people living with HIV, whereas robust prospective data addressing solid organ transplant recipients, patients receiving biological therapies, autoimmune diseases, and primary immunodeficiencies remain limited [12,20,37,38,39]. In addition, considerable heterogeneity persists across screening strategies, colposcopic management, and follow-up protocols, reflecting the lack of unified evidence-based approaches tailored to different immunological contexts [21,22,23,24].

Ongoing developments in molecular diagnostics, HPV genotyping, biomarker-based risk stratification, and artificial intelligence-assisted screening may contribute to more individualized and biologically informed management strategies in the future [35]. At the same time, expanding vaccination coverage and improving access to HPV-based screening programs remain central priorities for reducing the global burden of HPV-associated malignancies, particularly in low- and middle-income countries and vulnerable populations [1,2,3,4,5,6,7,8].

An additional area that deserves greater attention in future research and clinical practice is the psychosocial burden of HPV-related disease. Beyond its association with premalignant lesions and cancer, HPV infection may substantially affect health-related quality of life, emotional well-being, intimate relationships, body image, and sexual functioning [101,102,103,104,105,106]. Studies have shown that the diagnosis of HPV infection is frequently associated with anxiety, fear of cancer development, concerns regarding transmissibility, feelings of stigma and guilt, and uncertainty about future reproductive and sexual health. These effects may persist long after the initial diagnosis and can negatively influence relationship dynamics and overall quality of life. The psychosocial impact may be further amplified by the need for repeated surveillance, colposcopic examinations, biopsies, and excisional procedures, which have been associated with increased psychological distress and sexual dysfunction in some patients [101,102,103,104,105,106]. Despite growing recognition of these issues, patient-reported outcomes remain underrepresented in HPV research, particularly among immunosuppressed populations, in whom disease persistence, multifocal lower genital tract involvement, recurrence, and treatment burden are often greater. Future studies should incorporate validated measures of quality of life, mental health, and sexual function to better inform patient-centered care and supportive interventions across the spectrum of HPV-related disease [101,102,103,104,105,106]. Importantly, these patient-centered outcomes should be considered alongside traditional clinical and epidemiological endpoints when defining priorities for HPV prevention and control programs.

In this context, international initiatives such as the WHO cervical cancer elimination strategy represent an important milestone in global public health [100,107,108]. However, successful implementation of these goals requires adaptation to the clinical realities of immunosuppressed populations, in whom HPV-related disease follows a more aggressive and complex trajectory [6,24]. Bridging the gap between evolving scientific evidence, individualized clinical care, and equitable public health policies will be essential to optimize prevention, diagnosis, and treatment across the entire immunological spectrum.

## 12. Conclusions

Human papillomavirus infection should not be understood as a uniform clinical entity, but rather as a biologically dynamic spectrum shaped by the interaction between viral oncogenic potential and host immune competence. Although many principles related to HPV-associated disease are well established in gynecologic practice, this review highlights how immunological status substantially modifies disease presentation, multifocality, diagnostic complexity, therapeutic response, and risk of progression, particularly among immunosuppressed populations.

The evidence synthesized throughout this manuscript reinforces that management strategies traditionally developed for immunocompetent women are not always fully applicable to individuals with altered immune status, in whom persistent infection, broader lower genital tract involvement, and higher recurrence rates demand more individualized and proactive approaches. In this context, the integration of contemporary advances in molecular diagnostics, HPV-based screening, vaccination, and colposcopic assessment becomes essential to support risk-adapted clinical decision-making.

At the same time, current global efforts toward the elimination of cervical cancer underscore the importance of translating scientific advances into equitable and context-sensitive healthcare strategies. Bridging the gap between public health initiatives and the biological heterogeneity of HPV-related disease remains fundamental to improving prevention, diagnosis, and treatment outcomes across diverse and high-risk populations.

## Figures and Tables

**Table 1:** Clinical characteristics and management of HPV-related lesions by anatomical site.

Parameter	Cervix	Vagina	Vulva	Anal Canal
Clinical presentation	Asymptomatic (screen-detected)	Usually, asymptomatic	Visible lesions, pruritus, pain	Asymptomatic or bleeding, pain, pruritus
Typical lesions	CIN ^5^ 1–3	VaIN ^7^ 1–3	uVIN ^6^ 1–3	AIN ^4^ 1–3
Clinical suspicion	Abnormal cytology/HPV ^8^ DNA testing ^9^	Vaginal cytology	Visible lesion	Anal cytology/high-risk groups
Diagnosis	Colposcopy + biopsy	Colposcopy + biopsy	Inspection + biopsy	High-resolution anoscopy + biopsy
Indication for biopsy	Risk-based (ASCCP ^1^)	Suspicious lesions	Any doubtful lesion	Suspicious lesions or findings on HRA ^2^
Standard treatment	Excision (LEEP/conization)	Laser/ablation/excision	Excision, laser, or imiquimod	Ablation, excision, or topical therapies
High-risk lesion	HSIL ^3^ (CIN ^5^ 2/3)	HSIL ^3^ (VaIN ^7^ 2/3)	HSIL ^3^ (uVIN ^6^ 2/3)	HSIL ^3^ (AIN ^4^ 2/3)
Recurrence	5–25%	Moderate	High (up to 50%)	High (especially in HIV ^10^)
Risk of progression	Moderate–high (CIN 3 ^5^)	Lower than cervix	High	Elevated in high-risk groups
Impact of immunosuppression	High recurrence (40–60% in HIV ^10^)	High persistence	High multifocality and progression	High incidence, persistence, and progression

1. ASCCP: American Society for Colposcopy and Cervical Pathology; 2. HRA: high-resolution anoscopy; 3. HSIL: high-grade squamous intraepithelial lesion; 4. AIN: anal intraepithelial neoplasia; 5. CIN: cervical intraepithelial neoplasia; 6. uVIN: usual vulvar intraepithelial neoplasia; 7. VaIN: vaginal intraepithelial neoplasia; 8. HPV: human papillomavirus; 9. DNA: deoxyribonucleic acid; 10. HIV: human immunodeficiency virus.

**Table 2:** Colposcopic findings classification and their clinical implications in HPV-related disease.

Category	Finding	Clinical Description	Risk of HSIL	Recommended Management
Minor	Thin acetowhite epithelium	Faint, poorly demarcated whitening	Low	Follow-up or directed biopsy if persistent
	Fine punctation	Regular, fine capillary dots	Low	Follow-up/selective biopsy
	Fine mosaic	Delicate reticular pattern	Low	Follow-up/biopsy
Major	Dense acetowhite epithelium	Thick, opaque white epithelium	High	Biopsy
	Coarse punctation	Irregular, dilated vessels	High	Biopsy + consider treatment
	Coarse mosaic	Thick, irregular mosaic pattern	High	Biopsy
	Raised/irregular borders	Well-demarcated lesion with elevation	High	Biopsy
Suspicious for invasion	Atypical vessels	Irregular, bizarre, non-uniform vessels	Very high	Urgent biopsy and immediate oncologic referral
	Ulceration/necrosis	Surface disruption or tissue loss	Very high	Urgent biopsy and immediate oncologic referral

Adapted from: Bornstein et al. [48].

## Data Availability

No new data were created or analyzed in this study.
